# Coronal plane changes in fixed‐ versus mobile‐bearing unicompartmental knee replacements: Clinical and revision outcomes

**DOI:** 10.1002/jeo2.70221

**Published:** 2025-04-01

**Authors:** Enejd Veizi, Şahan Güven, Başak Sinem Sezgin, Christos Koutserimpas, Nevzat Arıcan, Ahmet Fırat, Çetin Işık, Murat Bozkurt

**Affiliations:** ^1^ Department of Orthopedics and Traumatology Faculty of Medicine, Ankara Yıldırım Beyazıt University Ankara Turkey; ^2^ Department of Orthopedics and Traumatology Ankara Bilkent City Hospital Ankara Turkey; ^3^ Department of Anatomy National and Kapodistrian University of Athens Athens Greece; ^4^ Department of Orthopedics and Traumatology VM Medical Park Ankara Hospital Ankara Turkey; ^5^ Çetin Işık Private Clinic Istanbul Turkey; ^6^ Department of Orthopedics and Traumatology Ankara Acıbadem Hospital Ankara Turkey

**Keywords:** arthroplasty, joint line obliquity, knee surgery, partial knee replacement, radiological measurements, unicompartmental knee replacement

## Abstract

**Purpose:**

To investigate the relationship between coronal plane changes after mobile‐ and fixed‐bearing (MB and FB) unicompartmental knee replacement (UKR) and clinical outcomes while also comparing revision rates and joint awareness between the two surgical modalities.

**Methods:**

Patients who operated between 2014 and 2017 with a UKR (FB or MB) were eligible for inclusion. Inclusion criteria were a minimum follow‐up of 5 years, presence of clinical outcome scores, Forgotten Joint Scores and radiological data (joint obliquity angles, tibial component alignment, angle between the medial and lateral joint lines, change in overall knee alignment, change in the medial proximal tibial angle). Outcome variables were compared between the MB and FB UKRs, and correlation analyses were performed to assess the effect of radiological changes on clinical and awareness scores. Two separate researchers performed all radiological measurements on direct radiographs.

**Results:**

Out of 229 patients, 197 were eligible for inclusion. Basic demographic data (mean age, sex, body mass index and follow‐up time) were comparable between the FB and MB groups. There were more revisions in the mobile bearing group (6.5% vs. 12.5%), though the results did not reach statistical significance. Clinical outcomes and joint awareness were similar in the two groups. Overall, the change in joint line obliquity or alignment was comparable between the groups.

**Conclusions:**

Clinical outcomes and joint awareness scores are similar in both fixed and mobile‐bearing UKRs. Revision is more frequent, though not statistically significant, following an MB UKR. Overall, change in knee alignment and medio‐lateral joint lines is similarly well tolerated in both implant modalities.

**Level of Evidence:**

Level III.

**Abbreviations**
BMI: body mass index
CPAK: Coronal Plane Alignment of the Knee
FB: fixed bearing
FJS: Forgotten Joint Score
ICC: intraclass correlation coefficient
JLO: joint line obliquity
KSS: Knee Society Score
MB: mobile bearing
MPTA: medial proximal tibial angle
OKS: Oxford Knee Score
PACS: picture and archiving system
TKR: total knee replacement
UKR: unicompartmental knee replacement

## INTRODUCTION

Unicompartmental knee replacement (UKR) seems to closely resemble native knee kinematics with good medium‐ and long‐term clinical results and survival [21, 27]. The procedure can be performed with a fixed‐bearing (FB) device or a mobile‐bearing (MB) device. A fixed‐bearing UKR uses a polyethylene insert that is fixed to the tibial component, explicitly allowing only flexion‐extension motion with limiting rotational movement. In contrast, a mobile‐bearing UKR features a rotating polyethylene insert, which can move in response to the tibial and femoral components, potentially mimicking natural knee movement more closely. Under optimal indications and with an intact anterior cruciate ligament (ACL) both fixed and mobile‐bearing UKRs have shown satisfactory results in restoring tibiofemoral kinematics allowing for the procedure to be extended to an increasingly younger patient population compared to the past decade [4]. Still, there is room for improvement as the UKRs have not been spared from the alignment debate with studies now showing promising results with kinematically aligned implants and restoration of the pre‐arthritic joint line obliquity [1, 11, 15].

Despite the recent shifting focus towards robotic‐assisted surgery and kinematic alignment in UKR, the vast majority of these surgeries are still performed manually and aim for mechanical axis restoration [7, 12]. While relatively easier to perform, the mechanical approach gives the surgeon the choice to use a mobile bearing or a fixed‐bearing UKR, as kinematically aligned UKR is typically performed with an FB implant [1], while the possibility of an MB UKR is still being explored [14, 24, 25, 26]. Mechanical alignment implies a perpendicular tibial cut to the mechanical axis of the tibia, often resulting in angular discrepancies between the medial and lateral tibial joint surfaces and eventually altering the patient's overall joint line obliquity (JLO).

Several studies have demonstrated comparable clinical results between MB and FB UKR implants [13, 28], but the design rationale of the MB implant would imply that discrepancies to the JLO would be better tolerated due to its higher degree of conformity mimicking the meniscal bearing surfaces. Bayoumi et al. [1] suggested that under correction of the arthritic deformity in FB UKRs led to worse clinical results and that overcorrection and restoration to native alignment had higher survival rates. On the other hand, several other authors have suggested that a tibial implantation in valgus, with an altered overall JLO, could have repercussions for the long‐term survivorship of the implant.

The objectives of this study were to investigate the relationship between coronal plane changes after MB and FB UKR and clinical outcomes while also comparing revision rates and joint awareness between the two surgical modalities. We hypothesized that no clinical differences would be present between the two surgical modalities in any of the planned measurements.

## METHODS

### Patient selection

This retrospective study was approved by the local ethical committee and patients gave their written and verbal consent for inclusion. Patients who operated between 2014 and 2017 with a medial UKR (fixed or mobile) were all eligible for inclusion. Inclusion criteria were a minimum of 5‐year follow‐up, presence of clinical outcome and radiological data. Patients were excluded from the study in case of missing or oblique direct radiographs and in case of death. Patients were operated on over the years by three different arthroplasty surgeons with more than 10 years of surgical experience in both FB and MB UKRs. The choice of the implant was made according to the surgeons' preference and hardware availability at the time of surgery. No clinical or radiographic criteria affected the choice of implant. The procedures were performed to restore the mechanical alignment and the overall hip–knee–ankle angle, with tibial cuts aimed perpendicularly to the mechanical axis of the tibia and femoral cuts tailored accordingly.

Demographic data regarding age, sex, side, body mass index (BMI) and revision surgery were collected from the hospital's digital record database. Direct radiographs were obtained from our centre's picture and archiving system (PACS). Data were analyzed in group comparison (FB vs. MB), while a combined analysis was performed to assess overall survival.

**Figure 1:**
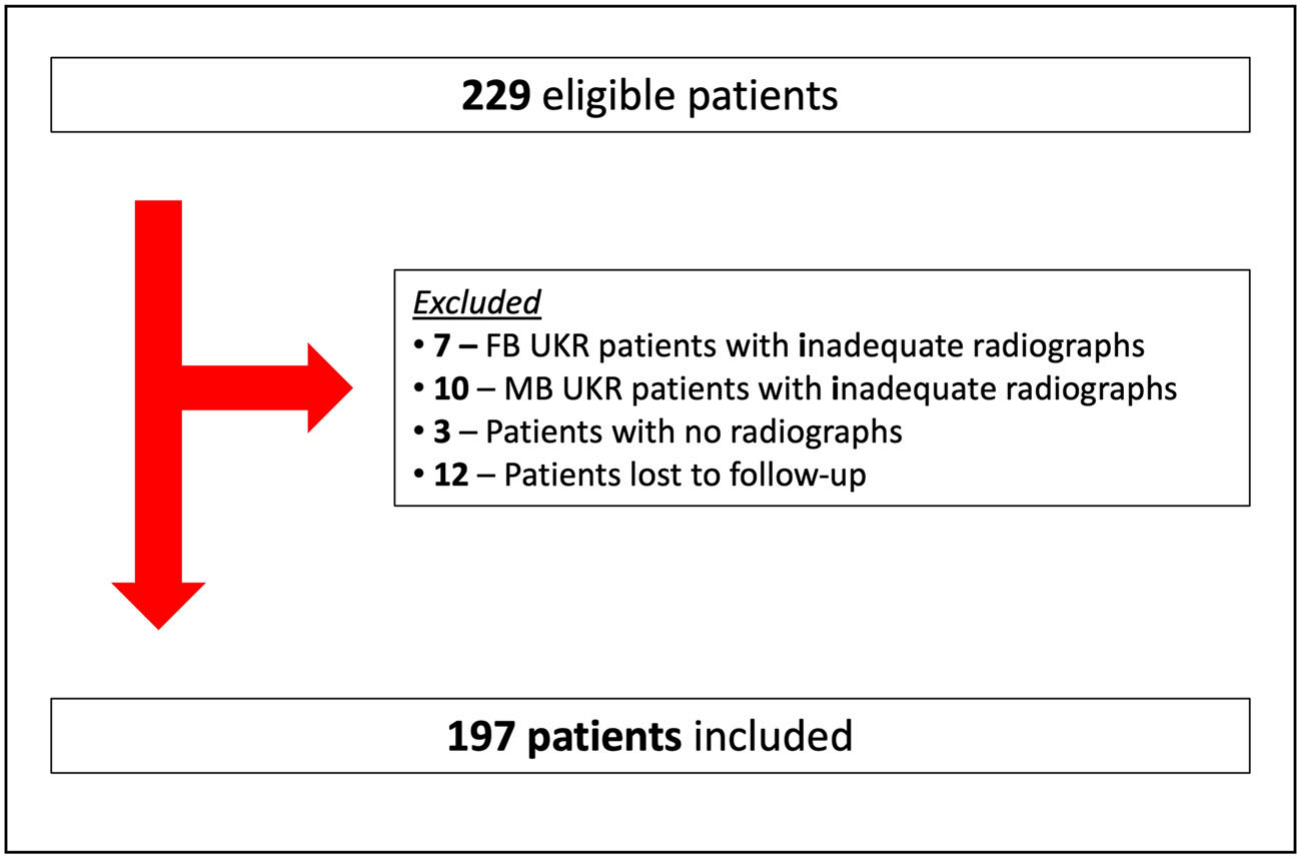
Flowchart of the study cohort. FB, fixed‐bearing; MB, mobile‐bearing; UKR, unicompartmental knee replacement.

Out of 229 patients eligible for inclusion, 197 were included in the final analysis (Figure 1).

### Radiological and clinical data

Preoperative and early post‐operative (Weeks 4–8) weight‐bearing antero‐posterior (AP) and lateral knee radiographs were used for evaluation. Antero‐posterior radiographs were taken from a distance of 100 cm, with the beam centred at the patella and including the distal femur and proximal tibia. Lateral radiographs were taken from the same distance and considered appropriate when femoral condylar superimposition was present, and the joint line was visible.

**Figure 2:**
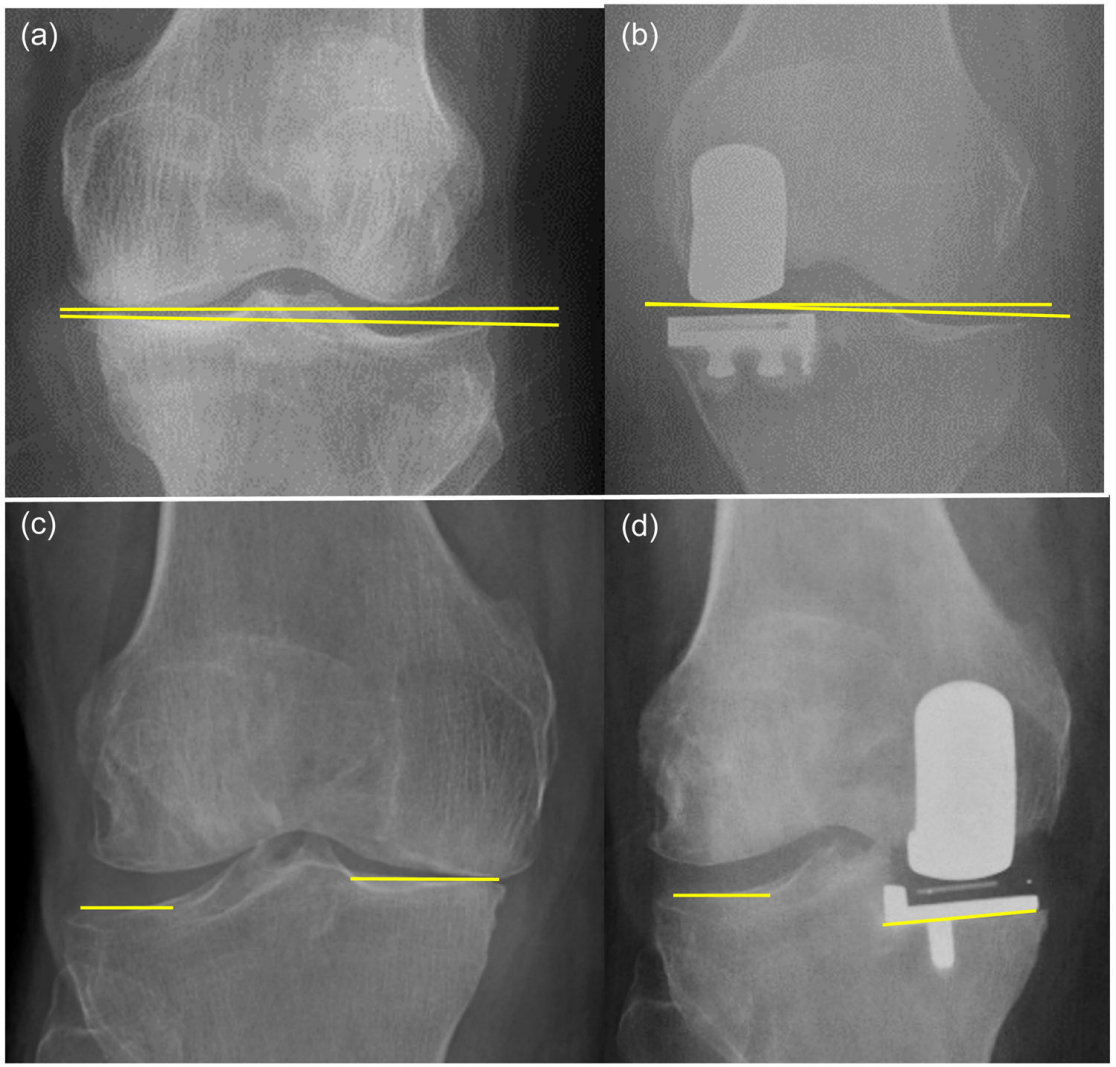
Measurement of joint line obliquity angle (a and b) and angle between the medial and lateral joint lines (c and d) on antero‐posterior direct knee radiographs.

**Figure 3:**
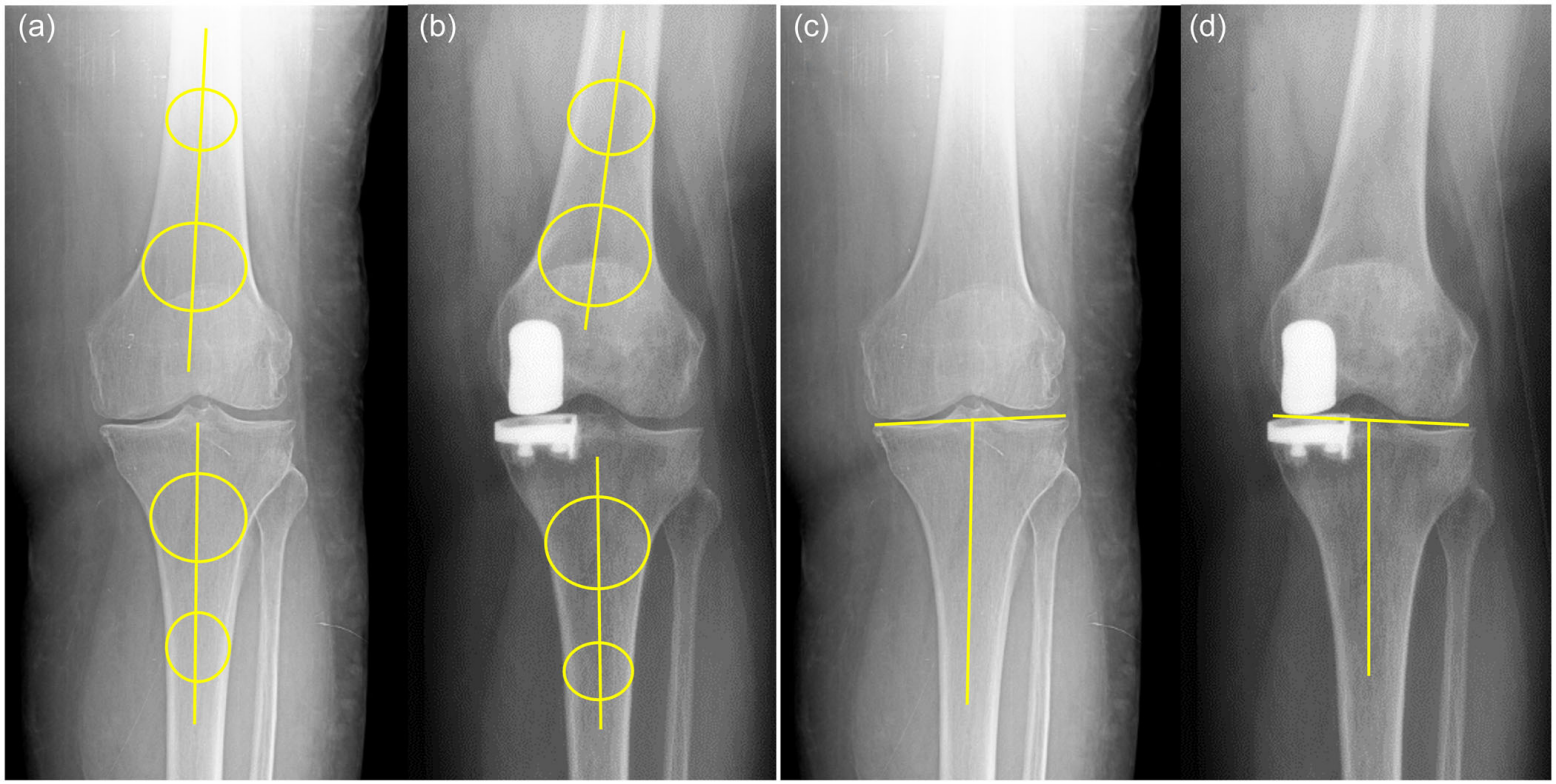
Measurement of knee joint alignment (a and b) and medial proximal tibial angle (c and d) on standing antero‐posterior direct knee radiographs.

The following measurement calculations were performed on the radiological images for all patients: preoperative Kellgren–Lawrence osteoarthritic grade, joint obliquity (JLO) angle (Figure 2a,b), tibial component alignment, the angle between the medial and lateral joint lines (Figure 2c,d), change in overall knee alignment (Figure 3a,b) and change in the medial proximal tibial angle (Figure 3c,d). The joint obliquity angle was measured using the Cobb angle function of the PACS and measuring the angle between a line tangent to both femoral condyles and a line drawn between the medial and lateral margins of the tibial plateau on the AP view. In post‐operative images, the line passing tangent to the femoral condyles and a line passing from the lateral condyle of the tibia and the distal border of the medial femoral component were used to calculate joint line obliquity.

**Figure 4:**
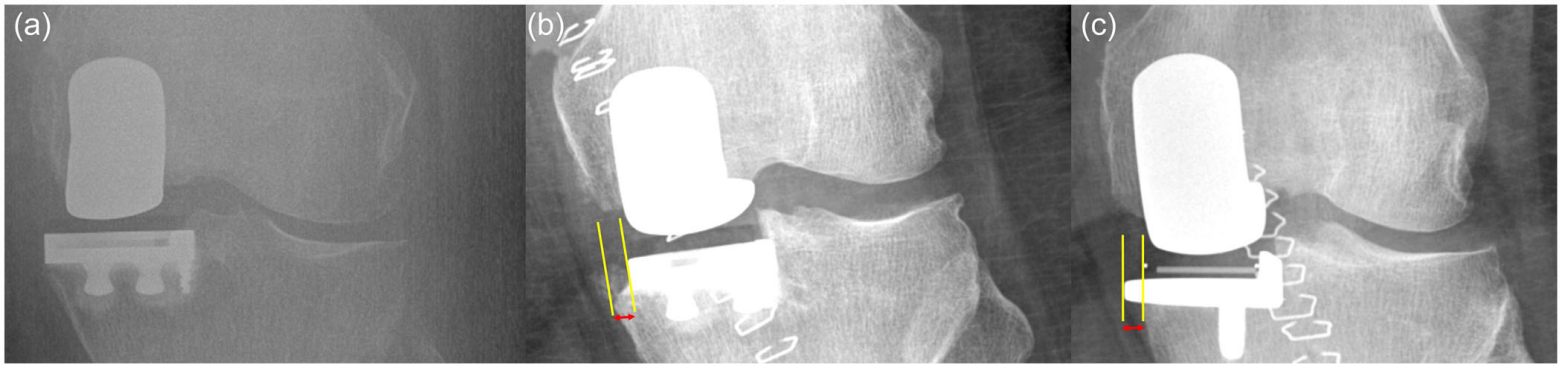
Radiographic depiction of a unicompartmental tibial component in a fit (a), under‐hang (b) and over‐hang (c) positions.

Tibial component alignment was defined as the angle of a line passing beneath the tibial component and the mechanical axis of the proximal tibia. The angle between the medial and the lateral joint surfaces was again measured using the Cobb function. Mid‐lines passing through the distal femur and the proximal tibia were used to measure the knee joint alignment, and the overall change was later calculated. Finally, the medial proximal tibial angle (MPTA) was measured preoperatively on native structures and post‐operatively as the line passing from the lateral condyle of the tibia and the upper distal border of the medial femoral component, as previously described by Micicoi et al. [18]. Changes between angles were calculated, and the data between the FB and MB UKRs were compared. Implant adequacy on the coronal plane was categorically assessed (noted as over‐hang, fit or under‐hang). Over‐hang and under‐hang were defined as a tibial baseplate protruding more than 2 mm or under‐hanging more than 2 mm on the coronal plane on direct AP radiographs (Figure 4). All measurements were performed once by two different researchers who were unaware of one another's results. The mean values of both measurements were used for the final analysis.

Clinical data regarding patient‐reported outcomes were gathered preoperatively and on the last follow‐up visit. Patients were evaluated with the Oxford Knee Score (OKS), Knee Society Score (KSS) and Forgotten Joint Score (FJS—post‐operative only) by an independent researcher, unaware of the UKR's modality. The data were compared between the two surgical options.

### Statistical analysis

Statistical analysis was performed using SPSS software v29.0 (IBM). Descriptive variables were presented as mean and standard deviations with median and minimal and maximal values. Categorical variables were presented as numbers and percentages. All study variables were assessed for normality distribution. Comparison between normally distributed variables was performed using the *t* test, while the Mann–Whitney *U* test was used for non‐parametric variables. The Pearson chi‐square test was used to compare categorical variables. Correlation analyses were performed to assess possible correlations between the changes in radiological angles and final clinical results. To assess interobserver reliability an intraclass correlation coefficient (ICC) was also calculated. A *p* < 0.05 was considered statistically significant.

## RESULTS

Baseline demographic data were comparable between the two groups. The mean age for the FB and MB groups was 58.1 ± 8.1 and 57.9 ± 6.4, respectively. Both cohorts were comprised of predominantly female patients (88.4% for the FB group and 86.8% for the MB group). The follow‐up period was also comparable within the groups. Interestingly, the FB implants were placed in optimal (fit) positions significantly more frequently compared to the MB group, where 25% of patients had an over‐hanging implant, and 8.7% had an implant with an under‐hang. Revision rates, while numerically higher in the MB group, were not statistically significant (Table 1).

**Table 1:** Demographic and basic radiological data of study groups.

	Fixed‐bearing UKA	Mobile‐bearing UKA	*p*
	(*n* = 92)	(*n* = 104)	
Age			0.737^a^
Mean ± SD	58.1 ± 8.1	57.9 ± 6.4	
Median (Min–Max)	57.0 (46.0–79.0)	57.0 (46.0–77.0)	
Side			0.568^b^
Right	47 (54.7%)	45 (49.5%)	
Left	39 (45.3%)	46 (50.5%)	
Sex			0.304^b^
Male	10 (11.6%)	12 (13.2%)	
Female	76 (88.4%)	79 (86.8%)	
BMI (kg/m^2^)			0.068^a^
Mean ± SD	33.4 ± 4.7	31.7 ± 4.5	
Median (Min–Max)	34.0 (22.3–45.8)	32.0 (21.5–41.0)	
Follow‐up time (months)			0.547^a^
Mean ± SD	92.6 ± 14.3	90.3 ± 10.5	
Median (Min–Max)	88.0 (73.0–118.0)	91.0 (73.0–119.0)	
Tibial component position			0.009^b^
Fit	78 (84.8%)	69 (66.3%)	
Over‐hang	12 (13.0%)	26 (25.0%)	
Under‐hang	2 (2.2%)	9 (8.7%)	
Kellgren–Lawrence			0.303^b^
1	6 (6.5%)	3 (2.9%)	
2	54 (58.7%)	70 (67.3%)	
3	29 (31.5%)	25 (24.0%)	
4	3 (3.3%)	6 (5.8%)	
Revision			0.057^b^
Yes	6 (6.5%)	13 (12.5%)	
No	86 (93.5%)	91 (87.5%)	

Abbreviations: BMI, body mass index; SD, standard deviation; UKR, unicompartmental knee replacement.

a Mann–Whitney *U* test.

^b^ Chi‐square test.

The mean change in joint line obliquity was 2.9 ± 2.2 for the FB group and 3.6 ± 1.8 for the MB group. An increase in medial to lateral joint line asymmetry was observed in both groups, as the mean change was 5.2 ± 4.0 for the FB and 4.8 ± 3.8 for the MB group. The positive character of the numbers delineates the overall valgus implantation of the tibial components, which can also be observed from the overall change in joint alignment (4.3 ± 3.0 and 4.1 ± 2.9, respectively). In the MB group, the changes in JLO were significantly higher compared to the FB group (*p* = 0.007). An ICC value of 0.877 was calculated for the study measurements, showing good inter‐observer reliability. Radiological data are presented in Table 2.

**Table 2:** Change between preoperative and post‐operative radiological parameters of study group.

	Fixed‐bearing UKA	Mobile‐bearing UKA	*p* a
	(*n* = 92)	(*n* = 104)	
ΔMPTA			0.061
Mean ± SD	4.0 ± 2.5	3.3 ± 2.1	
Median (Min–Max)	3.2 (0.0–10.6)	3.1 (0.1–8.6)	
ΔJLO			0.007
Mean ± SD	2.9 ± 2.2	3.6 ± 1.8	
Median (Min–Max)	2.6 (0.0–9.7)	3.5 (0.1–8.0)	
Δ of medio‐lateral joint line angle			0.507
Mean ± SD	5.2 ± 4.0	4.8 ± 3.8	
Median (Min–Max)	4.4 (0.1–17.4)	3.9 (0.3–17.9)	
Δ of knee joint alignment			0.472
Mean ± SD	4.3 ± 3.0	4.1 ± 2.9	
Median (Min–Max)	4.2 (0.0–11.3)	3.2 (0.0–11.7)	

Abbreviations: Δ, absolute change between post‐operative and preoperative values; JLO, joint line obliquity; MPTA, medial proximal tibial angle; SD, standard deviation; UKR, unicompartmental knee replacement.

a Mann–Whitney *U* test.

**Figure 5:**
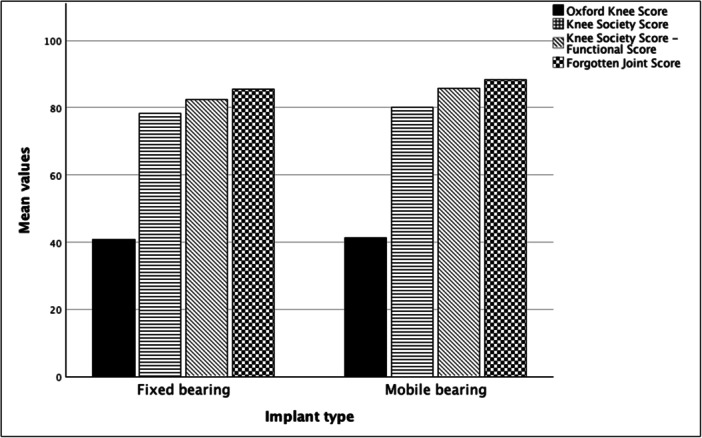
Clinical scores comparison between the study's groups. No significant difference was observed between the fixed‐ and mobile‐bearing unicompartmental knee replacements.

**Figure 6:**
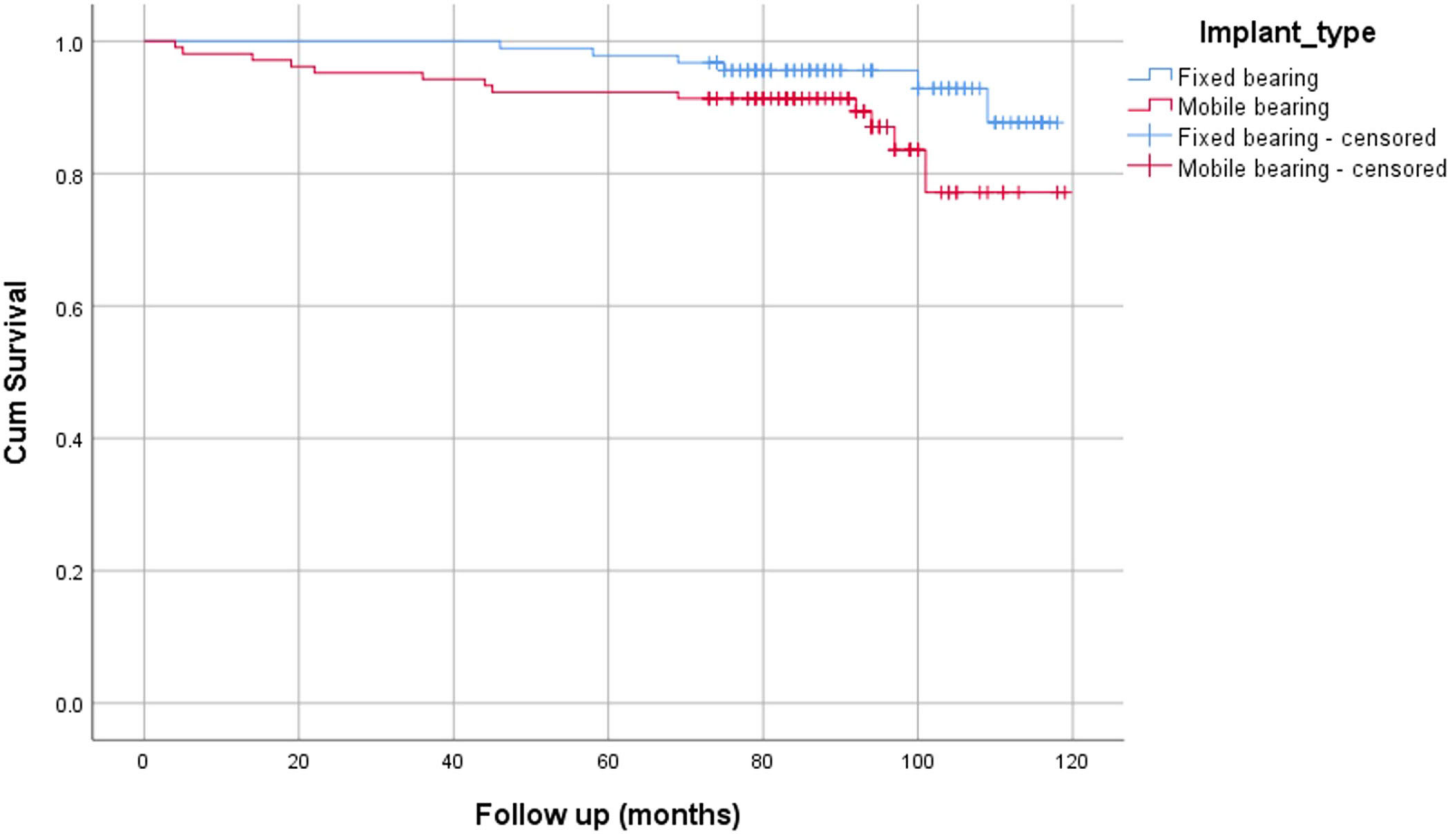
The Kaplan–Meier survival analysis showed no significant difference between the study groups at a mean follow‐up of 90 months (7.5 years).

**Figure 7:**
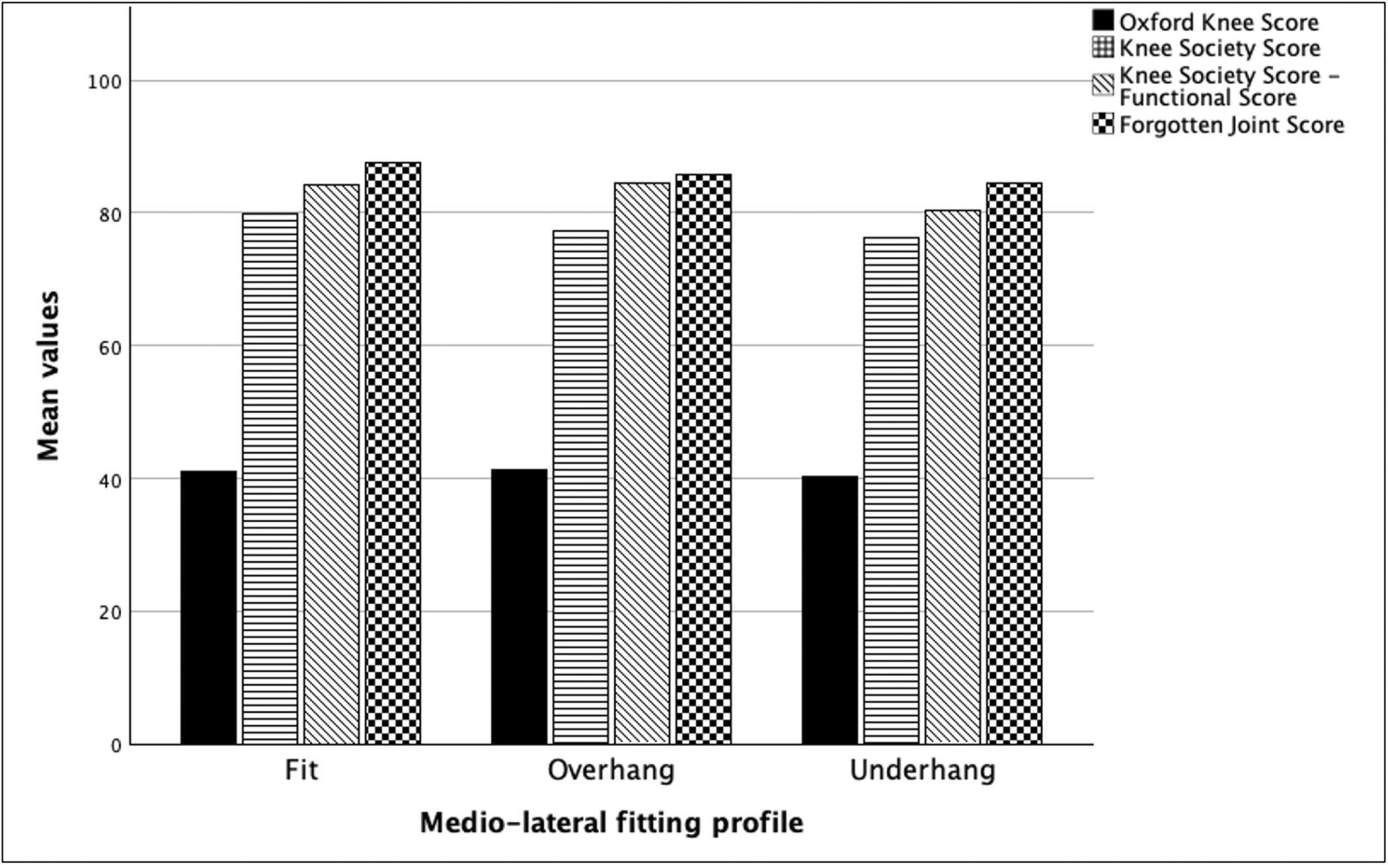
Subgroup analysis comparing clinical scores between the medio‐lateral fitting profile (fit, over‐hang and under‐hang). No statistically significant difference was found.

Clinical data on the final follow‐up, including the FJS, were comparable between the groups (all *p* values > 0.05) (Figure 5). Survival analysis revealed no statistical difference between the two implants (Figure 6) (log‐rank = 0.066, 93.5% vs. 87.5% survival). A correlation analysis was performed to evaluate the relationship between clinical scores and changes in angular values. No correlation was found between any of the measured changes and clinical scores. Also, a correlation analysis was performed to explore the relationship between coronal and sagittal implant position and clinical scores. Again, no significant correlation was found (Table 3). Finally, a subgroup analysis was performed to compare clinical scores between the ‘hanging’ status of the implant (fit, under‐hung or over‐hang). No statistically significant difference was found between these subgroups (all *p* values > 0.05) (Figure 7).

**Table 3:** Correlation analysis between clinical scores and changes in radiological measurements of the study cohort.

MB and FB UKR	OKS		KSS		KSS‐F	
	Correlation coefficient (*r*)	*p*	Correlation coefficient (*r*)	*p*	Correlation coefficient (*r*)	*p*
Δ Femoral‐tibial alignment	0.075	0.324	0.030	0.692	0.062	0.414
Δ JLO	−0.136	0.071	−0.011	0.889	−0.075	0.321
Δ ML JLO	0.124	0.100	−0.049	0.519	−0.030	0.689
Δ MPTA	0.025	0.744	−0.026	0.734	−0.068	0.368
Coronal tibia alignment	0.123	0.103	0.063	0.406	0.103	0.173

Abbreviations: Δ, absolute change between post‐operative and preoperative values; FB, fixed bearing; JLO, joint line obliquity; KSS, Knee Society Score; MB, mobile bearing; MPTA, medial proximal tibial angle; OKS, Oxford Knee Score; UKR, unicompartmental knee replacement.

## DISCUSSION

The most important finding of this study is that radiological changes of the coronal plane, including joint line obliquity, change of medial and lateral joint lines, medial proximal tibial angle and implant alignment, are consistently well tolerated in both FB and MB UKRs. This was also in line with our initial hypothesis. At a mean follow‐up of 7.5 years, overall implant survival is comparable between the two fixation modalities, and the medio‐lateral fitting profile does not significantly influence overall clinical results.

Overall survival and clinical results comparing FB and MB UKRs have been previously reported in the literature, with both implants showing excellent survival and acceptable clinical results [22]. Randomized trials are relatively rare since the implant modality is generally based on the surgeon's choice rather than clinical indication. Two studies from the past decades [3, 10] investigated the topic with no definitive conclusion, while Gilmour et al. [9] compared a robotic‐assisted FB‐UKR with a conventional MB‐UKR and found that overall clinical scores were comparable between the groups but suggested that younger and more active patients might benefit more from a robotic‐assisted procedure. More recently, Hariri et al. [13], in their matched‐pairs analysis, reported no overall clinical difference between the two implant modalities despite the MB groups having a wider degree of range of motion on the last follow‐up. This study yielded similar results, revealing that clinical outcomes at about 7.5 years of follow‐up were still comparable between the two implant modalities, and forgotten joint scores were similarly higher in both patient groups.

Implant survival has always been a special concern after a UKR due to their learning curve and the reluctance to abandon a ‘safer’ total knee replacement (TKR) option [5, 29]. Previous studies reported that almost 50% of knees requiring a UKR were subject to a TKR for various reasons, among which was the high revision rates reported after a UKR [20]. More recent research, on the other hand, shows a more diverse picture. Survival reports vary between 92.4% at 8 years [19], 95% at 10 years [17] and between 82% and 91% in the very long‐term follow‐up (15–20 years) [6, 23]. The results of the present study are consistent with the literature mentioned, showing a survival rate between 87% and 93% at a mean of 7.5 years of follow‐up. While revision was more frequent in the MB group, it failed to reach statistical significance.

Room for improvement is still being investigated with the concept of kinematically aligned UKR, with or without a robotic‐assisted system. Kim et al. [15] investigated the changes in the coronal plane after UKR and correlated them with the Coronal Plane Alignment of the Knee (CPAK) classification. They found that a change from the native subgroup to another in the mentioned classification yielded inferior clinical results, emphasizing the importance of maintaining or restoring the native coronal alignment. In contrast, Gill et al. [8] reported that a global residual varus was all it took to obtain overall better clinical results, independently of the femoral or tibial alignment. Finally, Micicoi et al. [18] reported the rates of restoration of the Cartier angle in UKR patients. This restoration is thought to correlate with a kinematically aligned component where the native tri‐dimensional articular surface orientation is restored. They concluded that functional results after medial UKR could be influenced by implant alignment in the coronal plane with slight clinical improvement in positioning the tibial implant close to the preoperative tibial deformity, rather than by restoring the Cartier angle. Despite the often‐conflicting results, there is a general consensus that a personalized alignment strategy for every single case would lead to better clinical results and longer survival rates after a UKR. The results of the present study show that the changes in alignment and JLO are similarly well tolerated in both MB and FB UKR modalities.

Overhanging of the tibial implant of more than two or three millimetres has historically been associated with increased post‐operative pain and collateral ligament irritation [2, 30, 31]. On the other hand, under‐hanging of the tibial implant has been well tolerated and sometimes encouraged [16, 31]. Wu et al. [31] specifically stated that the risk of post‐operative revision was increased seven‐fold in cases with a tibial overhang of ≥3 mm beyond the medial cortex of the tibial plateau. Interestingly, the present study exhibited a relatively high overhang incidence of 25% and 13% in MB and FB, respectively, but the hanging state seems to not be directly related to worse functional scores, independently of implant modality. This is contrary to the results of the literature, which state that a varus and overhang implant is expected to perform poorly. We believe this to be due to two factors: First, the overall values of our two cohorts lack outliers or extreme values. The range alignment of our final implant position is between 11° of varus and 8° of valgus in a very small number of patients. Second, all cases were performed by surgeons who had already completed their learning curve, leading to more accurate implant positioning in the majority of cases.

This study has some limitations. Firstly, it is a single‐centre, retrospective study and, therefore, inherently prone to bias. Nevertheless, it should be noted that most of the patients operated on during the study period were included, while the monocentric type ensures consistency in the surgical technique. Additionally, the study's radiological measurements, especially the knee joint alignment measurement, were performed on standing knee radiographs, which only allow for an approximation of the overall alignment and not panoramic (long‐axis) lower extremity views. This, furthermore, prohibited us from using the CPAK classification. Finally, sagittal plane measurements were not performed for this study since they were outside its objectives. While the categorical importance of ‘coronal knee alignment’ alone is being slowly replaced by the concept of ‘knee joint planes’, where all three alignment modalities (axial, sagittal and coronal) are taken into account during evaluation, our study explores only the coronal plane. Despite these limitations, our study showed that changes in the coronal plane do not affect the overall clinical results and joint awareness scores in patients operated with FB and MB UKRs.

## CONCLUSION

Clinical outcomes and joint awareness scores are similar in both FB and MB UKRs. Revision is more frequent, though not statistically significant, following an MB UKR. Overall, change in knee alignment and medio‐lateral joint lines is similarly well tolerated in both implant modalities.

## AUTHOR CONTRIBUTIONS

Enejd Veizi: Conceived the study idea and wrote part of the manuscript. Şahan Güven: Gathered data and contributed patients. Başak Sinem Sezgin: Wrote part of the manuscript and revised the manuscript. Christos Koutserimpas: Performed measurements and wrote part of the manuscript. Nevzat Arıcan: Performed measurements and statistical analysis. Ahmet Fırat: Wrote part of the revision and mentored the study. Çetin Işık: Gathered data and contributed to patients. Murat Bozkurt: Contributed to patients and mentored the study.

## CONFLICT OF INTEREST STATEMENT

The authors declare no conflict of interest.

## ETHICS STATEMENT

Ethical approval from the local ethics committee was obtained for this study. The approval was obtained from the 2nd Ethics Committee of the Ankara Bilkent City Hospital. IRB approval number is: E2‐24‐6119. All patients included in the study gave their verbal and written consent.

## Data Availability

Data regarding the present study will not be available in the public domain but can be provided to the Editor upon request.
